# Transcriptomic and metabolomic analyses reveal that bacteria promote plant defense during infection of soybean cyst nematode in soybean

**DOI:** 10.1186/s12870-018-1302-9

**Published:** 2018-05-11

**Authors:** Wenshu Kang, Xiaofeng Zhu, Yuanyuan Wang, Lijie Chen, Yuxi Duan

**Affiliations:** 1 Nematology Institute of Northern China, Shenyang Agricultural University, No.120 Dongling Road, Shenyang, 110866 China; 2 Institute of Biotechnology, Shenyang Agricultural University, No.120 Dongling Road, Shenyang, 110866 China

**Keywords:** Soybean, Soybean cyst nematode, *Bacillus simplex* strain Sneb545, Transcriptome, Metabolome, Nematicidal metabolites

## Abstract

**Background:**

Soybean cyst nematode (SCN) is the most devastating pathogen of soybean. Our previous study showed that the plant growth-promoting rhizobacterium *Bacillus simplex strain* Sneb545 promotes soybean resistance to SCN. Here, we conducted a combined metabolomic and transcriptomic analysis to gain information regarding the biological mechanism of defence enhancement against SCN in Sneb545-treated soybean. To this end, we compared the transcriptome and metabolome of Sneb545-treated and non-treated soybeans under SCN infection.

**Results:**

Transcriptomic analysis showed that 6792 gene transcripts were common in Sneb545-treated and non-treated soybeans. However, Sneb545-treated soybeans showed a higher concentration of various nematicidal metabolites, including 4-vinylphenol, methionine, piperine, and palmitic acid, than non-treated soybeans under SCN infection.

**Conclusions:**

Overall, our results validated and expanded the existing models regarding the co-regulation of gene expression and metabolites in plants, indicating the advantage of integrated system-oriented analysis.

**Electronic supplementary material:**

The online version of this article (10.1186/s12870-018-1302-9) contains supplementary material, which is available to authorized users.

## Background

Soybean is a crucial crop and a sustainable source of protein and oil worldwide. Soybean cyst nematode (SCN) is one of the most devastating pathogens in soybean that causes significant production losses. Possible countermeasures include resistant varieties, crop rotation, nematicides, and biological control [1]. Of these, chemical nematicides are generally not recommended for controlling SNC because of their non-selective nature, low efficiency, and high cost [2, 3]. In contrast, there are many advantages to using microbial control agents, including no impact to humans or other non-target organisms, decreased pesticide residues in food, conservation of natural enemies, and improvement of ecological diversity [4].

Plant growth-promoting rhizobacteria (PGPR) colonise the rhizosphere of many plant species and award favourable outcomes such as increased plant development and lessened susceptibility to diseases due to plant pathogenic fungi, bacteria, viruses, and nematodes [5]. PGPR compete for an ecological niche/substrate, and produce prohibitive allelochemicals, activating induced systemic resistance (ISR) in host plants against a broad spectrum of pathogens. Additionally, several PGPR strains protect plants against pathogen infection inducing systemic resistance [6]. Previous studies showed that bacteria of the genera *Pasteuria*, *Pseudomonas*, and *Bacillus* are highly effective as biological control agents against nematodes [7–9]. PGPR not only colonise the rhizosphere of many plants but also can induce disease resistance through seed priming. Microbial-mediated seed priming activates ISR against nematodes [10]. In cucumber, seed treatment with *Bacillus subtilis* strain GB03 enhances the plant growth and resistance against *Colletotrichum orbiculare*, *Pseudomonas syringae* pv. Lachrymans, and *Erwinia tracheiphila* [11], whereas that with *B. subtilis* S499 activates a systemic defence response against *Colletotrichum lagenarium* [12] in Arabidopsis, seed treatment with *B. subtilis* strain GB03 activates the signalling pathway of ethylene, independently of the salicylic acid or jasmonic acid signalling pathways, triggering defence responses against *Erwinia carotovora* subsp. [13]; and in soybean, seed treatment with *Bacillus simplex* strain Sneb545 induces resistance against SCN [14].

Metabolomic analysis has been successfully applied, providing valuable information on plant-pathogen interactions and system-wide variations in plant metabolism under pathogen infection and allowing the identification of compounds that play a pivotal role in plant innate immunity [15–22].

In this study, we combined transcriptomic and metabolomics analysis to identify differences in gene transcript levels and metabolic pathways between Sneb545-treated and non-treated soybeans under SCN infection. The resulting data might help to better understand the underlying molecular defence mechanisms of soybean and develop more effective biological control programs against SCN.

## Methods

### Plant materials, growth conditions and treatments

*B. simplex* strain Sneb545 was obtained from the Northern Nematode Institute, Shengyang Agriculture University, China [14] and grown on beef extract peptone agar medium at 25 °C for 48 h. Next, a single colony was selected to inoculate 100 ml beef extract peptone liquid medium and cultivated at 28 °C for 48 h on a rotary shaker at 150 rpm [23]. The fermentation broth was added in sterile water to a final optical density of 0.377 at 560 nm (1.0 × 10^9^ colony-forming unit ml^− 1^).

**Fig. 1:**
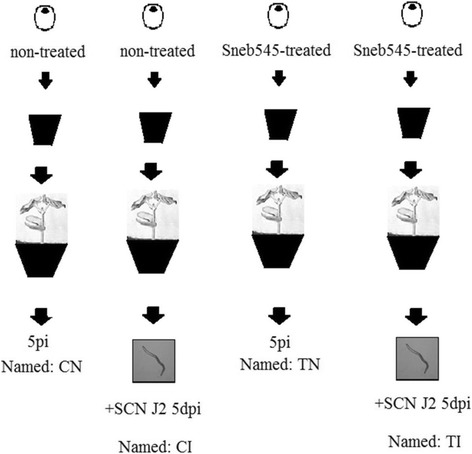
Graphical presentation of the laboratorial design. A float tray substrate was positioned in plastic pots reserved for two experiments (transcriptomic analysis and metabolomics analysis). The seeds were grown in a greenhouse with supplemental lighting (16 h light/8 h dark cycle, 26 ± 3 °C day/night). When the seeds were the two true leaves stage inoculated nematodes. There are three biological replicates in transcriptomic analysis and six biological replicates in metabolomics analysis. Per replicate of two plant roots is one sample. At 5 day post inoculation (dpi), roots samples were rapidly harvested, frozen in liquid N_2_, and stored at − 80 °C for further experiments

Seeds of the susceptible soybean cultivar Liao15 were surface-sterilised in 70% ethanol for 5 min and 2.1% sodium hypochlorite for 12 min and then, rinsed three times (10 min each) in sterile distilled water [24]. Next, the seeds were homogeneously coated with Sneb545 fermentation broth (Sneb545-treated soybeans) or with sterile distilled water (non-treated soybeans) and left to naturally dry at room temperature. A diagrammatic sketch of the experimental procedure is shown in Fig. 1. The seeds were sown in plastic pots (18 cm diameter) that contained a sterile soil mixture (soil: sand, 1:1) and placed in a greenhouse at 26 ± 3 °C day/night and a 16 h light/8 h dark cycle [23].

Mature females of SCN (*Heterodera glycines* Race 3) were harvested, and eggs were fixed as described previously [25]. Second-stage juveniles (J2) were collected to prepare J2 inoculum (1500 J2 ml^− 1^). Seedlings at the unifoliate stage were gently pulled out of the soil mixture and rinsed with water. Half of Sneb545-treated and non-treated soybeans were inoculated with 1 ml J2 inoculum that applied on the roots [24] to create four different treatments for analysis: Sneb545-treated soybeans infected with SCN (TI), non-treated soybeans infected with SCN (CI), Sneb545-treated soybeans not infected with SCN (TN), and non-treated soybeans not infected with SCN (CN). Four biological replicates were used for transcriptomic analysis, whereas seven biological replicates for metabolomics analysis. At 5 d post infection (dpi), two roots samples per plant were harvested, immediately frozen in liquid nitrogen, and stored at − 80 °C until analysis. One replicate was stained with acid fuchsine for visualizing the infection [26]. Three biological replicates were used for transcriptomic analysis, whereas six biological replicates for metabolomics analysis.

### Nematode infection assay

In TI and CI, SCN development from J2 to third-stage juveniles (J3) was detected as described previously [26]. Data were analysed to identify any significant differences at *p* < 0.05 *n* = 5 using SPSS software 17.0.

### RNA extraction, library construction, and sequencing

Frozen root tissue was milled to powder in a mortar with liquid nitrogen. Total RNA isolation was performed using RNeasy Plant Mini Kit (Qiagen, Hilden, Germany). RNA quality was tested by 1% agarose gel electrophoresis, whereas RNA purity using NanoPhotometer (IMPLEN, Westlake Village, CA, USA). RNA concentration was measured using Qubit 2.0 Fluorometer (Life Technologies, Carlsbad, CA, USA) with Qubit RNA Assay Kit. RNA integrity was assessed using Bioanalyzer 2100 (Agilent Technologies, Santa Clara, CA, USA) with RNA Nano 6000 Assay Kit (Aglient Technologies). The library construction and sequencing were performed at Novogene Corporation (http://novogene.com/index.php). The construction and sequencing of soybean cDNA libraries were performed at Novegene Company (Beijing, China; http://www.novogene.com).

### Data processing assembly and annotation

Raw reads of fastq format were processed using in-house perl scripts. Clean reads were obtained by removing those containing adapter, or ploy-N and as well as those of low-quality and used in all subsequent analyses. The Q20, Q30, and GC content of clean data were also calculated.

Reference genome and gene model annotation files were downloaded from (ftp://ftp.ncbi.nlm.nih.gov/genomes/all/GCF/000/004/515/GCF_000004515.4_Glycine_max_v2.0/GCF_000004515.4_Glycine_max_v2.0_genomic.fna.gz). The index of the reference genome was constructed using Bowtie 2.2.3 [27] and paired-end clean reads were aligned to the reference genome using TopHat 2.0.12 [28] which can generate a database of splice junctions based on the gene model annotation file, providing better mapping results than other non-splice mapping tools.

HTSeq 0.6.1 was used to calculate the number of reads mapped to each gene. The expression level of each gene was estimated by the number of fragments per kilobase of transcript sequence per million base pairs (FPKM) that calculated based on the gene length and number of mapped reads [29].

Differential expression analysis of two groups (TI vs TN and CI vs CN; three biological replicates per group) was performed using DESeq for R (DOI: 10.18129/B9.bioc.DESeq). KOBAS 2.0 [30] was used to test the statistical enrichment of differentially expressed genes (DEGs) in Kyto Encyclopaedia of Genes and Genomes (KEGG) pathways. The total number of genes involved in a pathway was counted and then the *p*-value was calculated using hypergeometric distribution [31].

### Quantitative real-time (qRT)-PCR validation

To validate RNA-seq results, the expression patterns of 21 randomly selected genes from various functional categories and regulation patterns were analysed by qRT-PCR at five different time points post infection (Additional file 1: Table S1). Three biological replicates were collected independently and immediately frozen in liquid nitrogen. Total RNA isolation was performed as described above, and 1 μg of total RNA was reverse-transcribed using PrimerScript First-Strand cDNA Synthesis Kit (Takara, Dalian, China). qRT-PCR was performed in a 25 μl reaction mixture containing 12.5 μl 2 × SYBR Master Premix ExTaq (Takara), 1 μl cDNA template (1:5 dilution), and 1 ul of each forward and reverse primer for the selected gene of interest. All primers were designed based on cDNAs using Primer Premier 5.0 (Additional file 1: Table S1). Three biological and three technical relipcates per sample were analysed using Bio-Rad Real-Time System (BioRad, Hercules, CA, USA) based on the 2^-ΔΔCT^ method [32]. qRT-PCR conditions were 95 °C for 2 min followed 40 cycles at 95 °C for 5 s, 60 °C for 30 s and 65 °C for 5 s. Actin11 (GeneBank: 209698678) was used as a reference gene.

### Metabolomic analysis

Take 50 mg sample into the 2 mL EP tubes, extracted with 0.4 mL extraction liquid (Vmethanol:VH2O = 3: 1), add 20 μL of Adonitol (CAS#: 488–81-3, ≥99%, Sigma, China) 2 mg/mL stock in dH2O as internal standard. Homogenized in ball millSamples for 4 min at 40 Hz used Grinding mill (JXFSTPRP-24, Shanghai, China), then ultrasound treated for 5 min (incubated in ice water). Centrifuge for 15 min at 12000 rpm, 4 °C. Transferred the supernatant (0.35 mL) into a fresh 2 mL gas chromatography-mass spectroscopy (GC/-MS glass vial, take 9 μL from each sample and pooling as QC sample. Dry the extracts in a vacuum concentrator without heating. Add 20 μL Methoxyamination hydrochloride (20 mg/mL in pyridine), incubation for 30 min at 80 °C. Add 30 μL of the BSTFA regent (1% TMCS, *v*/v) to the sample aliquots, incubated for 2 h at 70 °C. Add 7 μL FAMEs (Standard mixture of fatty acid methyl esters, C8-C16: 1 mg/mL; C18-C24: 0.5 mg/mL in chloroform) to the QC sample it cooling to the room temperature. Mix well for GC-MS analysis.

Samples for gas chromatography-mass spectroscopy (GC-MS) were prepared as described previously [33], and analysis was performed using Agilent 7890 gas chromatograph system (Agilent Technologies) coupled with Pegasus HT time-of-flight mass spectrometer (LECO, St. Joseph, MI, USA). The system utilised a DB-5MS capillary column coated with 5% diphenyl and cross-linked with 95% dimethylpolysiloxane (30 m × 250 μm inner diameter, 0.25 μm film thickness; J & W Scientific, Folsom, CA, USA). A 1 μl aliquot was injected in splitless mode. Helium was used as the carrier gas, the front inlet purge flow was 3 ml min^− 1^, and the gas flow rate through the column was 1 ml min^− 1^. The initial temperature was maintained at 50 °C for 1 min, then raised to 300 °C at a rate of 10 °C min^− 1^, and maintained at 300 °C for 8 min. The injection, transfer line, and ion source temperature was 280 °C, 270 °C, and 220 °C, respectively. The energy in the electron impact mode was − 70 eV. Mass spectrometry data were acquired in a full-scan mode with an m/z range of 50–500 at a rate of 20 spectra s^− 1^ after a solvent delay of 460 s.

### Data analysis

The GC/MS raw data were processed by Chroma TOF4.3X (LECO) using LECO-Fiehn Rtx5 database [34]. The normalised data were used for orthogonal partial least squares discriminant analysis (OPLS-DA) by SIMCA 14.1 (Umea AB, Umea, Sweden).

### Statistical and metabolite-transcript network analysis

One-way analysis of variance (ANOVA) in conjunction with Student’s t-test was performed to determine significant differences between groups at *p* < 0.05. Principal component analysis (PCA) of the total transcriptomic dataset was performed in R (R Core Team). Transcriptome and metabolome datasets were combined to create a general metabolite-transcript network centred on genes related to phenylpropanoid biosynthesis, cysteine and methionine metabolism, tropane, piperidine and pyridine alkaloid biosynthesis, unsaturated fatty acid biosynthesis, and fatty acid metabolism according to KEGG (http://www.kegg.jp/). Structure diagrams were constructed in Microsoft Word 2010 (Microsoft Corporation, Redmond, WA, USA).

### Nematode mortality assessment

The mortality rate of J2 at different concentrations (500 μg ml^− 1^, 600 μg ml^− 1^, and 1000 μg ml^− 1^) of 4-vinylphenol, L-methionine, piperine, and palmitic acid was assessed in vitro as described previously [35] and calculated as follows: ((live J2 prior to exposure − live J2 at 24 or 48 h post exposure)/live J2 prior to exposure) × 100. All compounds were of analytical grade and purchased from Sigma-Aldrich (St Louis, MO, USA), except for piperine that purchased from Jianglai (Shanghai, China). Each treatment was repeated in triplicate, and the experiment was repeated four times. L-methionine was dissolved in water, palmitic acid and 4-vinylphenol in 3% Tween 80 and 4% ethanol, whereas piperine in 3% Tween 80 and 4% methanol. The respective diluents were used as controls. All statistical analyses were performed in SPSS software 17.0.

## Results

### Assessment of Sneb545-induced resistance

**Fig. 2:**
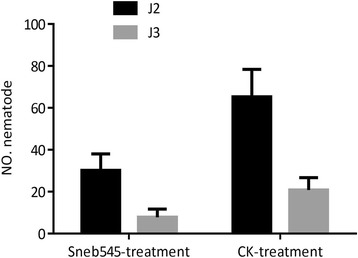
Number of different developmental stages soybean cyst nematode in roots of Sneb545 treatment and control treatment soybean roots at 5 day post inoculation (dpi). Each value represents the mean of five replicated±the standard error. Significant differences between Sneb545 treatment and control treatment soybean roots were identified with *t* test calculated with SPSS Base Ver.17.0, the significance threshold set at *P* < 0.05

The ability of Sneb545 to induce soybean resistance to SCN has been recorded previously [14]. Compared with CI, the number of nematodes in TI was lower by 72.63%, whereas the ratio of nematodes in the soil adjacent to soybean roots was lower by 70.63%. Except for reducing the penetration rate of nematodes, Sneb545 also inhibited the growth of nematodes in soybean roots (Fig. 2).

### Identification of DEGS

In total, four separate un-normalised cDNA libraries were generated for each treatment (TI, CI, TN, and CN) that produced 3.929–5.546 million clean reads per library. Of these, 94.31–99.46% of bases had a Q ≥ 20, whereas 87.29–98.14% of bases a Q ≥ 30 (Additional file 2: Table S2). A total of 39.290–55.458 million reads from each library (80.85–92.75% of total reads) was mapped to the reference genome (Additional file 3: Table S3). All the raw data was submitted to Sequence Read Archive database (SAMN07305253).

**Fig. 3:**
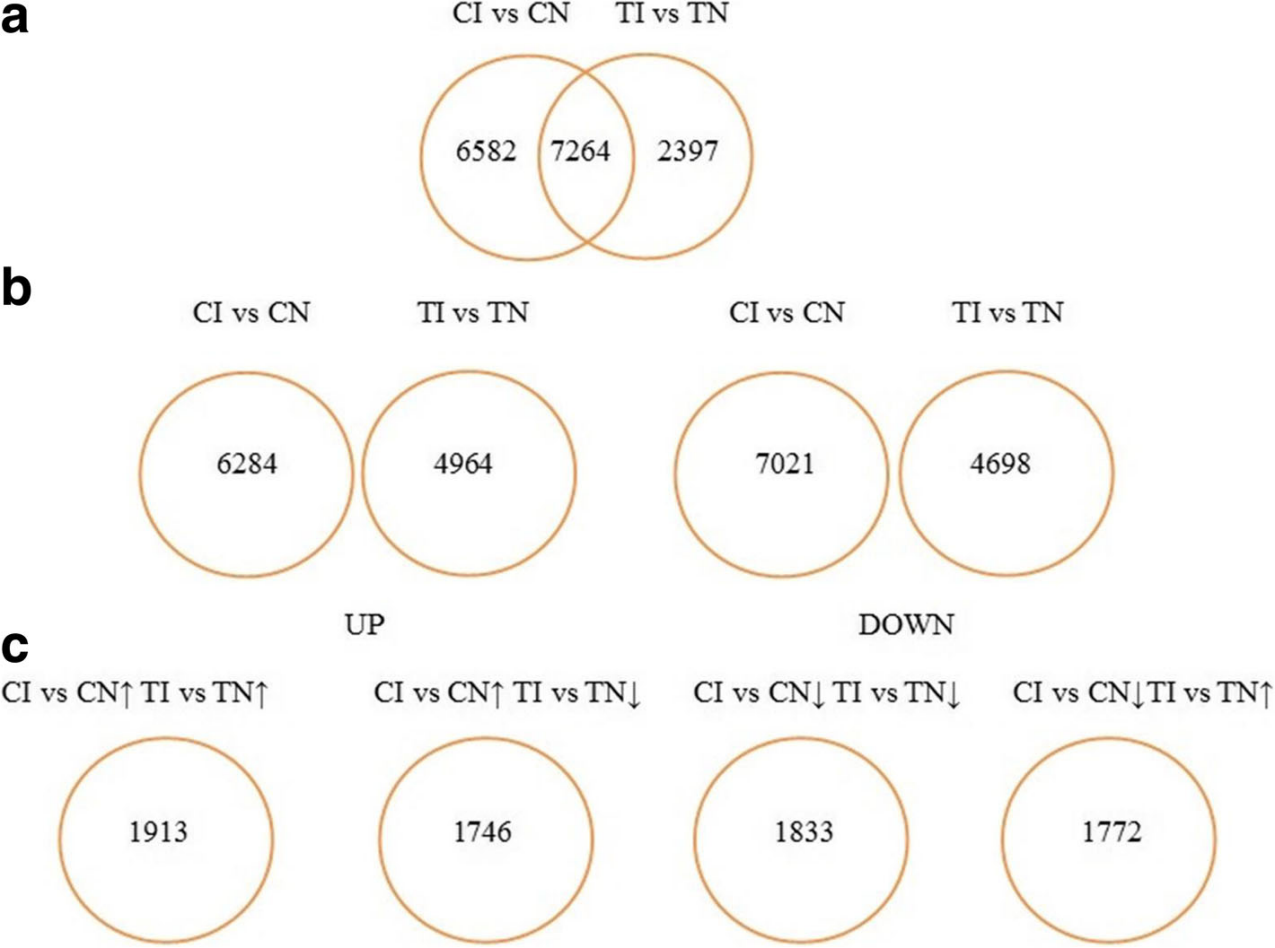
Venn diagrams showing commonality uniqueness of the constitutively-related genes between non-treated and Sneb545-treated soybeans infected with SCN. In total, 16,243 genes were constitutively regulated in the different treatment soybeans (*p* < 0.05). (Sneb545-treated soybeans infected with SCN (TI), non-treated soybeans infected with SCN (CI), Sneb545-treated soybeans not infected with SCN (TN), and non-treated soybeans not infected with SCN (CN)). **a** Transcripts represented different accumulation. **b** Transcripts represented up and down accumulation in different comparable group. **c** Transcripts represented that overlapped up and down accumulation

FPKM values were used to measure the expression level of each assembled transcript sequence in different samples, and a padj<0.05 was applied to identify changes in transcript abundance in the TI vs. TN and CI vs. CN group. As shown in Fig. 3 9661 DEGs were identified in the TI vs. TN group and 13,846 DEGs in the CI vs. CN group. Of these, 4694 and 6284 DEGs were up-regulated, whereas 4698 and 7021 DEGs were down-regulated in the TI vs. TN and CI vs CN group, respectively. Additionally, 1913 DEGs were up-regulated and 1833 down-regulated in both groups, 1746 DEGs were down-regulated in TI vs. TN group but up-regulated in CI vs. CN group, whereas 1772 DEGs were up-regulated in the TI vs.TN group but down-regulated in the CI vs.CN group. The top 20 pathways for TI vs.TN group and CI vs.CN group are based on KEGG analyses are shown in Additional file 4: Figure S1 and Additional file 5: Figure S2.

### qRT-PCR

We conducted qRT-PCR to validate the RNA-seq data and to analyze the gene expression changes of randomly selected genes at the time point 5dpi. Although the expression levels of selected genes were different between RNA-seq and qRT-PCR, the trend of expression were the same (Additional file 1: Table S1). These genes were all down-regulated in CI vs CN group and up-regulated in TI vs TN group.

### Effect of Sneb545 on secondary metabolites and integrated metabolite networks

**Fig. 4:**
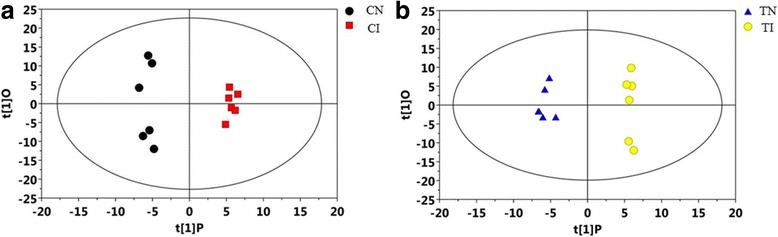
OPLS-DA model analysis was performed using the most diverse sample. Sneb545-treated soybeans infected with SCN (TI), non-treated soybeans infected with SCN (CI), Sneb545-treated soybeans not infected with SCN (TN), non-treated soybeans not infected with SCN (CN). **a** OPLS-DA in CI vs CN group. **b** OPLS-DA in TI vs TN group

As shown in Fig. 4, the variable importance in the projection (VIP) statistic of the first principal component of OPLS-DA model (threshold > 1), together with the *p*-value of the Student’s t-test (threshold < 0.05) were used for selecting significant variables responsible for group separation. The level of 4-vinylphenol, L-methionine, and palmitic acid was lower in CI than in CN, but almost the same in TI and TN. However, the level of piperine was the highest in TI, followed by that in other group. Based on KEGG, the four metabolites were enriched to 20 different metabolic pathways (Additional file 6: Table S5).

### Comprehensive networks of transcripts and metabolites

Using the combined transcriptomic and metabolomic data, we aimed to identify changes in KEGG pathways involving phenylpropanoid biosynthesis, cysteine and L-methionine metabolism, tropane, piperidine, and pyridine alkaloid biosynthesis, unsaturated fatty acid biosynthesis, and fatty acid metabolism (Additional file 7: Table S6).

**Fig. 5:**
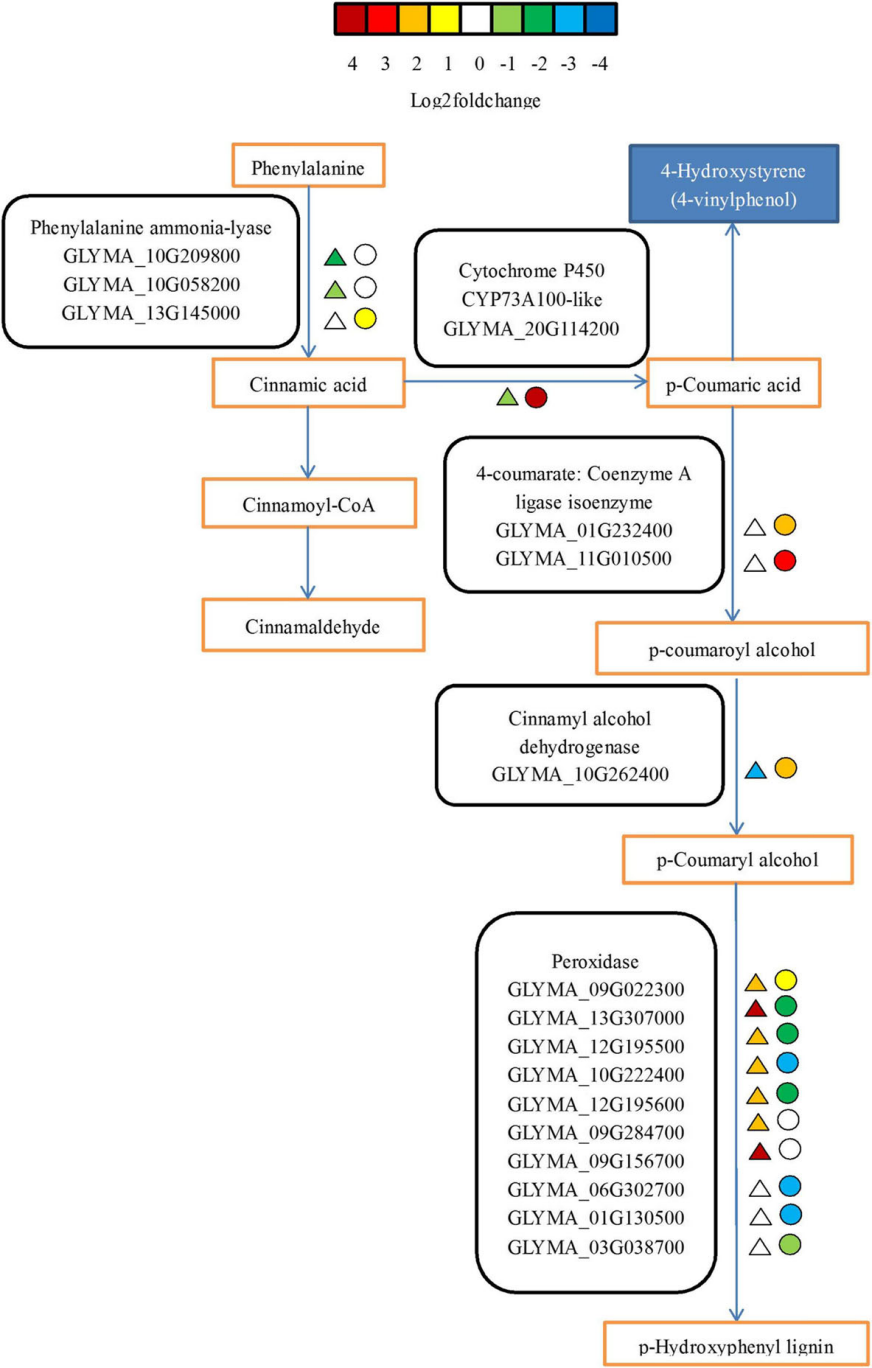
Modulation of phenylpropanoid biosynthesis pathway under Sneb545-treated and non-treated soybean roots infected with SCN compared with not infected with SCN. Log2foldchange levels of different gene expression are presented at left triangle box (non-treated soybean roots infected with SCN compared with non-treated soybean roots not infected with SCN, CI vs CN), and at right square box (Sneb545-treated soybean roots infected with SCN compared with Sneb545-treated soybean roots not infected with SCN, TI vs TN). Green and red boxes indicate down- or up-regulation of the genes

In the phenylpropanoid biosynthesis pathway, a cytochrome P450 CYP73A100 gene (GLYMA_20G114200) was up-regulated in the TI vs. TN group and down-regulated in the CI vs. CN group. Of three phenylalanine ammonia-lyase (PAL) genes, two (GLYMA_10G209800 and GLYMA_10G058200) were down-regulated in the CI vs. CN group and suppressed in the TI vs. TN group, whereas one (GLYMA_13G145000) was suppressed in the CI vs. CN group and up-regulated in the TI vs. TN group. Two 4-coumarate-CoA ligase (4CL) genes (GLYMA_01G232400 and GLYMA_11G010500) were suppressed in the CI vs. CN group and down-regulated in the TI vs. TN group. Of 10 peroxidase (POD) genes, five (GLYMA_09G022300, GLYMA_13G307000, GLYMA_12G195500, GLYMA_10G222400, and GLYMA_12G195600) were up-regulated in the CI vs. CN group and down-regulated in the TI vs. TN group; two (GLYMA_09G284700, and GLYMA_09G156700) were up-regulated in the CI vs. CN group and suppressed in the TI vs. TN group; whereas three (GLYMA_06G302700, GLYMA_01G130500, GLYMA_03G038700) were suppressed in the CI vs. CN group and down-regulated in the TI vs. TN group. Additionally, one cinnamyl alcohol dehydrogenase (CAD) gene (GLYMA_10G262400) was up-regulated in the CI vs. CN group and down-regulated in the TI vs. TN group (Fig. 5 and Additional file 7: Table S6).

**Fig. 6:**
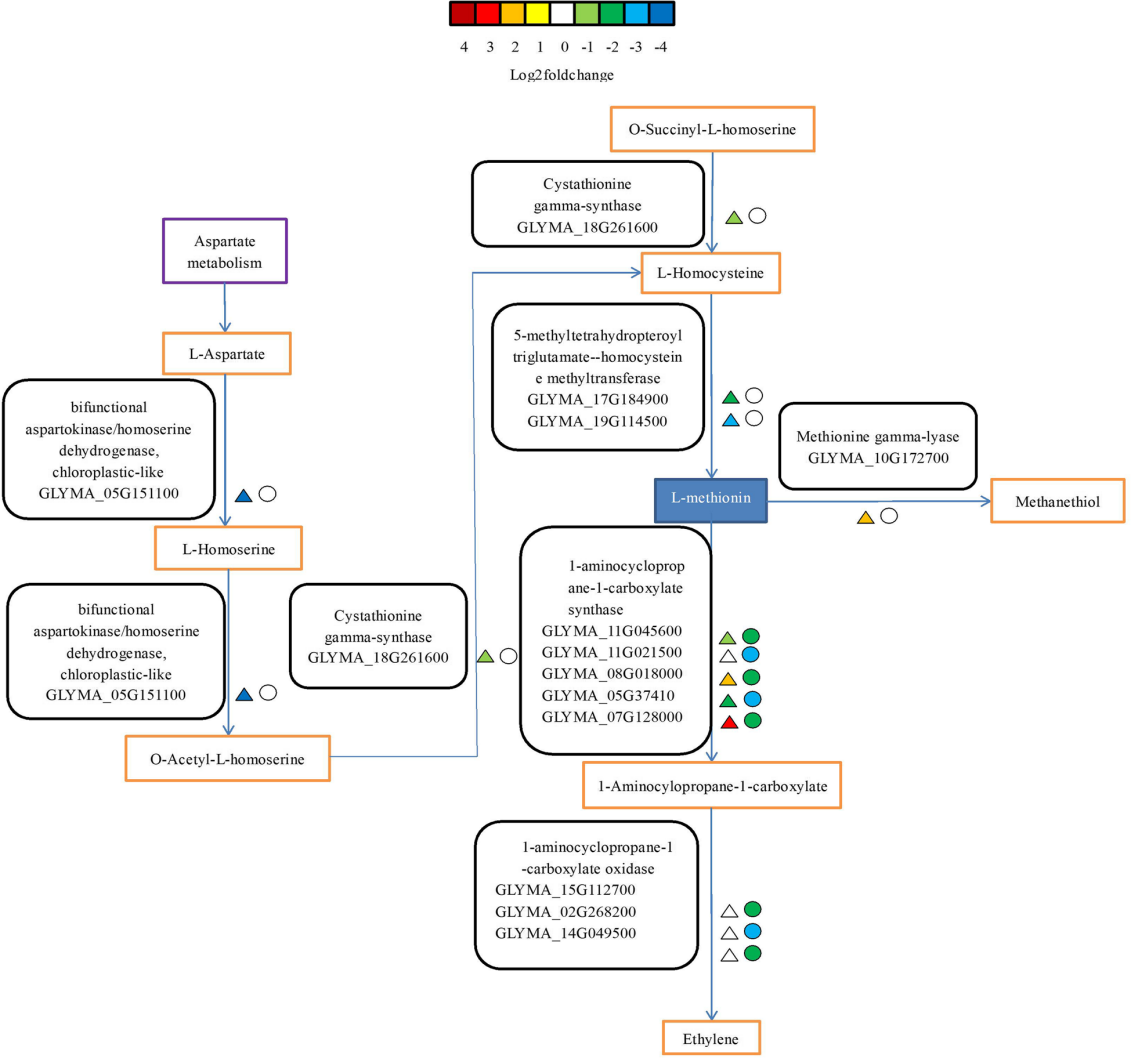
Modulation of cysteine and methionine metabolism pathway under Sneb545-treated and non-treated soybean roots infected with SCN compared with not infected with SCN. Log2foldchange levels of different gene expression are presented at left triangle box (non-treated soybean roots infected with SCN compared with non-treated soybean roots not infected with SCN, CI vs CN), and at right square box (Sneb545-treated soybean roots infected with SCN compared with Sneb545-treated soybean roots not infected with SCN, TI vs TN). Green and red boxes indicate down- or up-regulation of the genes

In the cysteine and methionine metabolism pathway, a methionine gamma-lyase (MGL) gene (GLYMA_10G172700) was up-regulated in the CI vs. CN group and suppressed in the TI vs. TN group. Five 1-aminocyclopropane-1-carboxylate synthase (ACS) genes (GLYMA_11G045600, GLYMA_11G021500, GLYMA_08G018000, GLYMA_05G37410, and GLYMA_07G128000) were down-regulated in both groups, but the downward fold change was higher in the TI vs. TN group. Three 1-aminocyclopropane 1-carboxylate oxidase (ACO) genes (GLYMA_15G112700, GLYMA_02G268200, and GLYMA_14G049500) were suppressed in the CI vs. CN group and down-regulated in the TI vs. TN group. Additionally, one bifunctional enzyme aspartokinase-homoserine dehydrogenase (bifunctional AK-HSDH) gene (GLYMA_05G151100), one cystathionine gamma-synthase (CGS) gene (GLYMA_18G261600), and two 5-methyltetrahydropteroyltriglutamate-homocysteine methyltransferase (MET6) genes (GLYMA_17G184900, and GLYMA_19G114500) were down-regulated in the CI vs. CN group and suppressed in the TI vs. TN group (Fig. 6 and Additional file 7: Table S6).

**Fig. 7:**
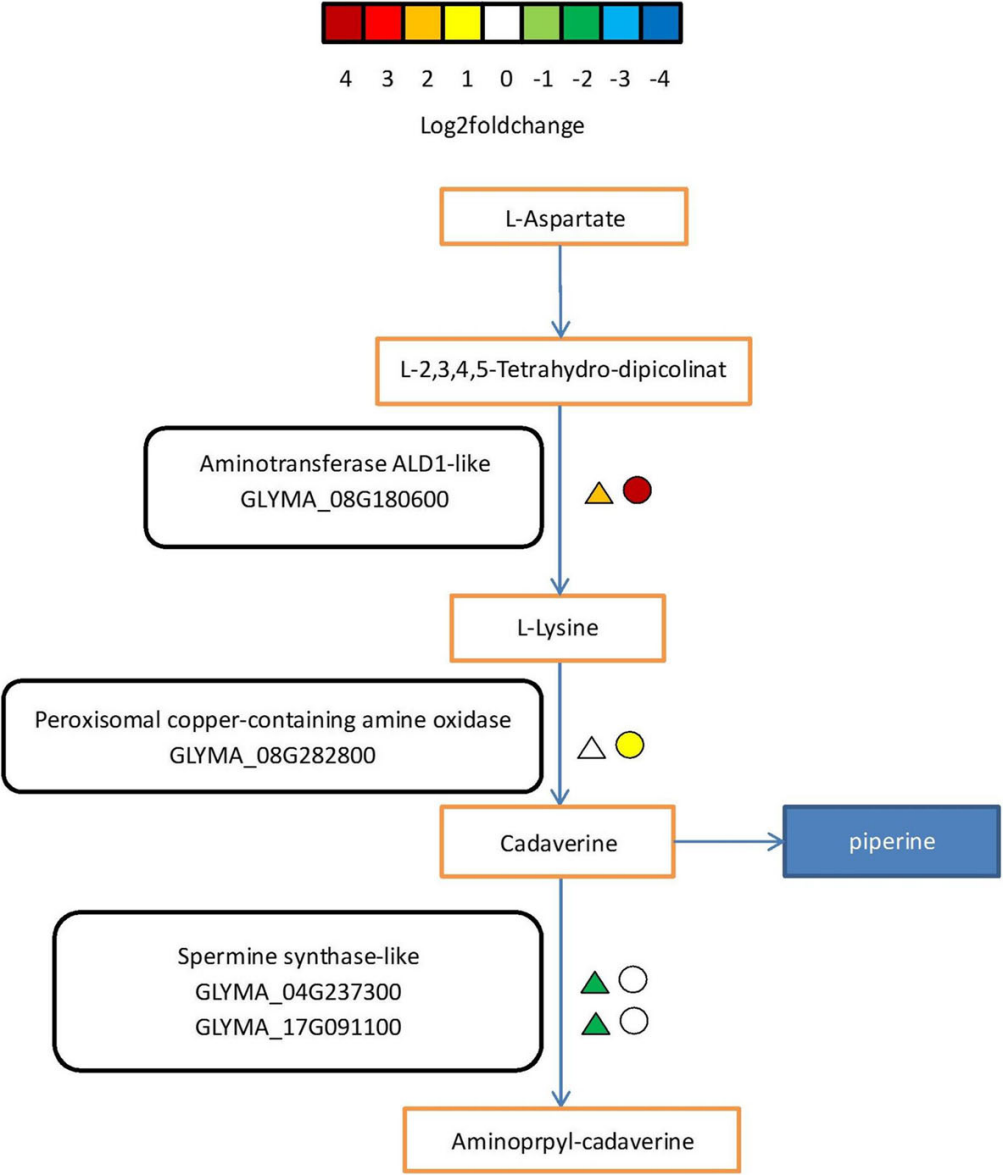
Modulation of tropane, piperidine and pyridine alkaloid biosynthesis metabolism pathway under Sneb545-treated and non-treated soybean roots infected with SCN compared with not infected with SCN. Log2foldchange levels of different gene expression are presented at left triangle box (non-treated soybean roots infected with SCN compared with non-treated soybean roots not infected with SCN, CI vs CN), and at right square box (Sneb545-treated soybean roots infected with SCN compared with Sneb545-treated soybean roots not infected with SCN, TI vs TN). Green and red boxes indicate down- or up-regulation of the genes

In the tropane, piperidine, and pyridine alkaloid biosynthesis pathway, one peroxisomal copper-containing amine oxidase (CuAO) gene (GLYMA_08G282800) was suppressed in the CI vs. CN group and up-regulated in the TI vs. TN group; two spermine synthase genes were up-regulated in the TI vs. TN group and suppressed in CI vs. CN group; and one aminotransferase (ALD1) gene was up-regulated in both groups, but the upward fold change was higher in the TI vs. TN group (Fig. 7 and Additional file 7: Table S6).

**Fig. 8:**
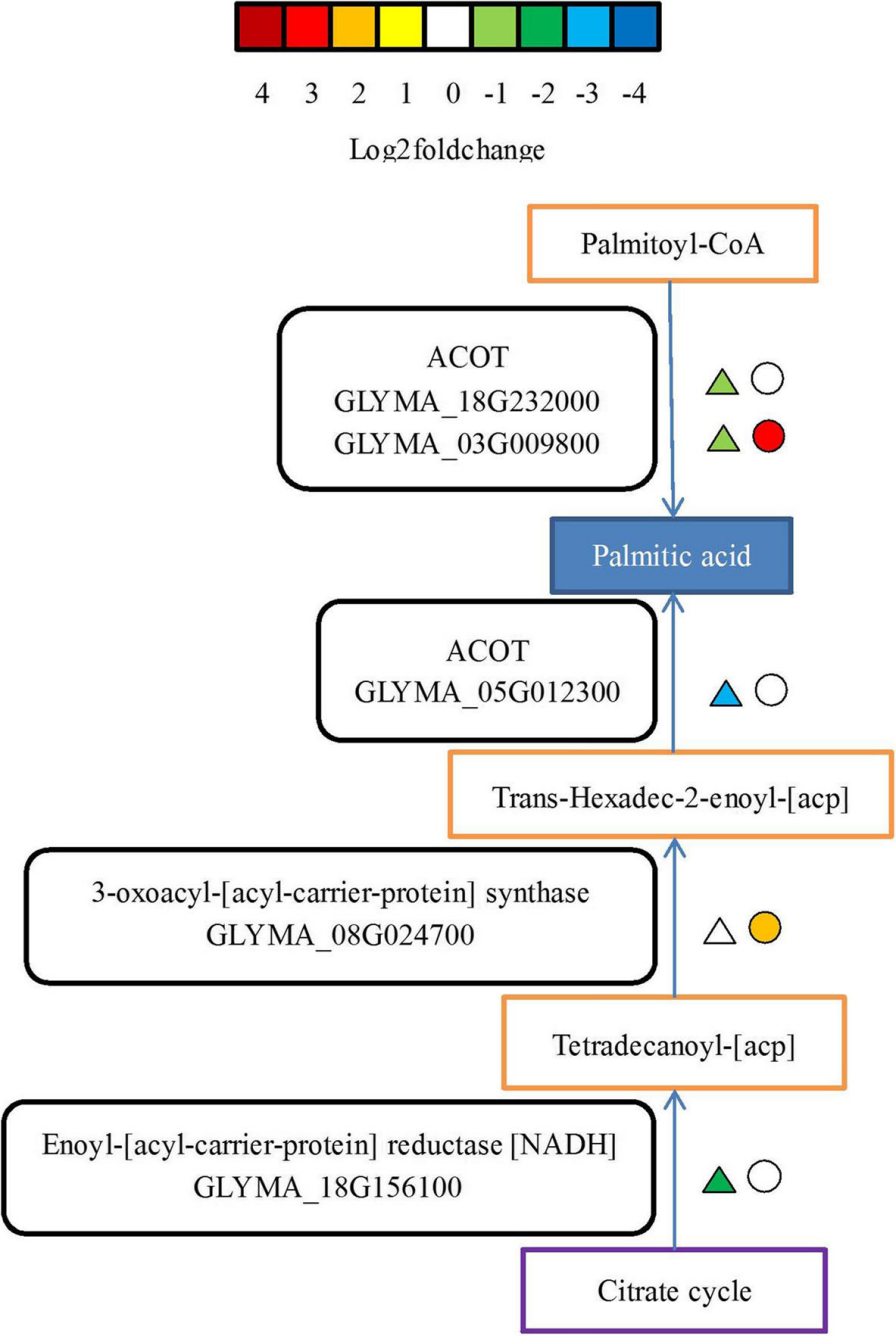
Modulation of biosynthesis of unsaturated fatty acids and Fatty acid metabolism pathway under Sneb545-treated and non-treated soybean roots infected with SCN compared with not infected with SCN. Log2foldchange levels of different gene expression are presented at left triangle box (non-treated soybean roots infected with SCN compared with non-treated soybean roots not infected with SCN, CI vs CN), and at right square box (Sneb545-treated soybean roots infected with SCN compared with Sneb545-treated soybean roots not infected with SCN, TI vs TN). Green and red boxes indicate down- or up-regulation of the genes

In the unsaturated fatty acid biosynthesis and fatty acid metabolism pathway, one 3-oxoacyl-[acyl-carrier-protein] synthase gene (GLYMA_08G024700) was suppressed in the CI vs. CN group and up-regulated in the TI vs. TN group. Of four acyl-acyl-carrier (ACP) thioesterase genes, three (GLYMA_18G232000, GLYMA_03G009800, and GLYMA_05G012300) were down-regulated in the CI vs. CN group and one (GLYMA_03G009800) was up-regulated in the TI vs. TN group. Additionally, one enoyl-[acyl-carrier-protein] reductase (ENR) gene (GLYMA_18G156100) was down-regulated in the CI vs. CN group and suppressed in the TI vs. TN group (Fig. 8 and Additional file 7: Table S6).

### Nematode mortality assessment

Nematode mortality assessment indicated that 4-vinylphenol, L-methionine, piperine and palmitic acid might be effective nematicidal compounds (Table 1). At 48 h post treatment with L-methionine (500 μg ml^− 1^), palmitic acid (600 μg ml^− 1^), 4-vinylphenol (1000 μg ml^− 1^) and piperine (600 μg ml^− 1^), the corrected nematode mortality rates were 82.8, 77.8, 87.9, and 79.9%, respectively.

**Table 1:** Effects of metabolites from commercial sources at 48 h corrected death. rate on soybean cyst nematode second-stage juveniles (J2)

Treatment	48 h Corrected J2 death rate (%)
L-methionine^a^ (500 μg/ml)	82.8 ± 0.01
Palmitic acid^b^ (600 μg/ml)	77.8 ± 0.01
4-vinylphenol^b^ (600 μg/ml)	87.9 ± 0.02
Piperine^c^ (1000 μg/ml)	79.9 ± 0.02

Means of twelve replicates with the same letter do not differ significantly (*P* ≤ 0.05) (the mean of corrected death rate ± SE)

^a^L-methionine was dissolved in water and the water as the control

^b^Palmitic acid and 4-vinylphenol were dissolved in 3% Tween 80 and 4% ethanol, at the same time 3% Tween 80 and 4% ethanol as the control

^c^Piperine was dissolved in 3% Tween 80 and 4% methanol, at the same time 3% Tween 80 and 4% methanol as the control

## Discussion

Information on the effectiveness of *B. simplex* as a biocontrol agent against SCN is limited. Our previous study showed that *B. simplex* strain Sneb545 increases soybean resistance to SCN. Thus, our objective in the present study was to conduct a combined metabolomic and transcriptomic analysis to gain information regarding the biological mechanism of soybean defence enhancement to SCN via Sneb545 treatment. The results showed that Sneb545 modulated the accumulation of 4-vinylphenol, L-methionine, piperine, and palmitic acid in soybean roots under SCN infection. Our nematode mortality assessment showed that 4-vinylphenol has nematicidal activity against SCN at a minimum effective concentration of 1000 μg ml^− 1^ (Table 1). These results were in agreement with those reported in a previous study and revealed the nematicidal activity of 4-vinylphenol against *Caenorhabditis elegans* in three Rosaceae species at a minimum effective concentration of 600 μg ml^− 1^ [36]. Based on our metabolomic data, the levels of 4-vinylphenol were higher in TI than in CI (Additional file 8: Table S4), indicating that Sneb545 induced the accumulation of 4-vinylphenol, improving soybean resistance against SCN.

In the phenylpropanoid biosynthesis pathway, PAL plays an important role in plant defence [37–39]; CAD is an indicator of lignin biosynthesis and plays an important role in plant responses to biotic and abiotic stresses [40–43]; 4CL is often associated with induced defence [43]; whereas POD functions as a scavenger of reactive oxygen species typically produced as an early plant resistance response against a wide range of pathogens and may be involved in blocking oxidant-mediated programmed cell death [44, 45]. Previous microarray studies have indicated that SCN infection of susceptible soybeans leads to the suppression of PAL and 4CL genes and the up-regulation of CAD and POD genes [46–51]. Similar changes in gene expression were identified in the CI vs. CN group but not in the TI vs. TN group, suggesting that Sneb545 might induce soybean resistance to SCN.

In the cysteine and methionine pathway, many genes play important roles in plant defence against nematode infection. Our nematode mortality assessment showed that methionine has nematicidal activity at a minimum effective concentration of 500 μg ml^− 1^ (Table 1). These results were in agreement with those reported in a previous study and revealed the nematicidal activity of methionine against *Belonolaimus longicaudatus* Rau (Belonolaimidae) and *Mesocriconema ornata* Raski (Criconematidae) [52, 53]. Both transcriptomic and metabolomic analysis demonstrated that Sneb545 induced the accumulation of methionine under SCN infection. MGL that is a key gene in sulfur amino acid degradation [54] was suppressed in TI, inhibiting L-methionine degradation. The precursor of ethylene, 1-aminocyclopropatie-1-carboxylate (ACC), is produced by the conversion of S-adenosyl-Met by ACS [55], whereas ACO is responsible for the final stage of ethylene production in higher plants [56]. Arabidopsis mutants that overproduced ethylene or wild-type plants treated with ACC showed an increased number of female nematodes in the roots [57]. Similarly, a soybean mutant sensitive to ethylene showed a 50% decrease in the number of SCN in the roots [58]. In the present study, ACS and ACO were down-regulated in TN, improving soybean resistance against SCN. Additionally, bifunctional AK-HSDH I and II that catalyse L-aspartic acid, which synthesises is L-methionine [59]. MET6 that converts homocysteine to L-methionine [60]; and CGS that participates in the biosynthesis of methionine [61, 62] were up-regulated in TN, enhancing the accumulation of L-methionine and consequently, the resistance against SCN.

In the present study, the nematode mortality assessment showed that piperine has nematicidal activity at a minimum effective concentration of 600 μg ml^− 1^ (Table 1). These results were in agreement with those reported in a previous study and revealed the nematicidal activity of piperine against *Meloidogyne javanica* by inhibiting the function of glutathione S-transferase [63, 64]. In the tryptophan, piperidine, and pyridine alkaloid pathway, CuAO catalyses the oxidation of primary alcohol groups of amino acids to the corresponding amino aldehydes with the concomitant production of H2O2 and NH3 [65]. CuAO shows differential localization in the apoplast and peroxisomes, but in both locations, it contributes to modulating plant defence against different types of stress [66]. In pea, the resistant cultivar P665 showed increased levels of peroxisomal CuAO under *Meloidogyne pinodes* infection compared with those of the susceptible cultivar Messire [67]. Spermidine synthase is a key enzyme involved in polyamine biosynthesis as a specific 10A06 interactor. Previous studies showed that SPDS2 promoter is strongly activated in the nematode-induced syncytia, and transgenic plants overexpressing SPDS2 have enhanced susceptibility to *Heterodera schachtii* [68]. ALD1 encodes aminotransferases that act on an overlapping set of amino acids in vitro [69] and may be a key component in disease resistance by the regulation of nematicidal metabolites [70, 71]. In the present study, CuAO and ALD1 were up-regulated, whereas SPDS was suppressed in the TI vs. TN group, improving soybean resistance against SCN (Additional file 8: Table S4).

The nematode mortality assessment in the present study showed that palmitic acid has nematicidal activity at a minimum effective concentration of 600 μg ml^− 1^ (Table 1). Similarly, a previous study showed that a nematicidal fatty acid mixture obtained from *Hericium coralloides* exhibited both repellent and nematicidal effects on *C. elegans* [72]. Palmitic acid identified in the benzene root extracts of *Iris japonica* also showed nematicidal properties [73]. In the unsaturated fatty acid biosynthesis and fatty acid metabolism pathways, acyl-CoA thioesterase (ACOT) shows the highest rate of hydrolysis with palmitoyl-CoA and forms palmitic acid [74]; acyl-ACP thioesterase (FATA) is a chain-length-determining enzyme in the de novo biosynthesis of fatty acids in plants; 3-oxoacyl-[acyl-carrier-protein] synthase facilitates the condensation reaction of fatty-acid synthesis by increasing an acyl acceptor of two carbons from malonyl-ACP, resulting in the elongation from C-10 to unsaturated C-16 and C-18 fatty acids [75]; and ENR stimulates a key regulatory step in fatty acid biosynthesis [76]. In the present study, ACOT, FATA, 3-oxoacyl-[acyl-carrier-protein] synthase, and ENR showed different expression profiles in the TI vs. TN and CI vs. CN groups and revealed that Sneb545 induced the accumulation of palmitic acid in the former group improving its resistance against SCN.

The identification of a parasite by the roots can induce a hypersensitive reaction, leading to the discharge of nematicidal compounds such as alkaloids, terpenes, phenols, and amino acids [77–79]. Here, we showed that Sneb545 could induce the production of nematicidal compounds that inhibit the growth of nematodes in the soybean root.

## Conclusion

To better understand the Sneb545-induced soybean resistance to SCN, we performed a combined transcriptomic and metabolomic analysis to identify any differences in gene expression and secondary metabolites between Sneb545-treated and non-treated soybeans under SCN infection. Our results showed that phenylpropanoid biosynthesis, cysteine and methionine metabolism, tropane, piperidine, and pyridine alkaloid biosynthesis, unsaturated fatty acid biosynthesis, and fatty acid metabolism might participate in the Sneb545-induced soybean response to SCN. Additionally, we revealed that Sneb545-treated soybeans accumulated four nematicidal metabolites (4-vinylphenol, L-methionine, piperine, and palmitic acid) that inhibited SCN development. However, further studies are needed to evaluate the effectiveness of accumulated nematicidal metabolite application as a biological control measure against SCN.

## Additional files

Additional file 1: **Table S1.** Comparison of expression profiles of random selected 21 genes by RNA-seq and qRT-PCR data. Twenty one genes were randomly selected from the genes differentially regulated, and examined for their expression at 5 days post-inoculation using. The results were the average from three biological replicates. (XLSX 12 kb)

Additional file 2: **Table S2.** Statistics of DEG sequencing. The data quality assessment of sample sequencing output is detailed in the table. Raw reads: statistics of original sequence data. Clean data/clean reads: after filtering the raw data, the remaining data of the low quality data is removed. Subsequent analyses are based on clean data. Q20, Q30: calculate the percentage of bases of Phred value greater than 20 and 30, respectively. GC content: the total amount of base G and C is calculated as a percentage of the total bases. (XLSX 10 kb)

Additional file 3: **Table S3.**Statistics of reads mapped back to the reference transcriptome. Total reads: the sequencing sequence has been filtered by the sequencing data (Clean data). Total mapped: the number of sequences that can be located on the genome; n general, if there is no pollution and the reference genome is appropriate, the percentage of this data is greater than 70%. (XLSX 9 kb)

Additional file 4: **Figure S1.** Differentially expressed genes enriched KEGG pathway in CI vs CN group. (JPG 172 kb)

Additional file 5: **Figure S2.** Differentially expressed genes enriched KEGG pathway in TI vs TN group. (JPG 176 kb)

Additional file 6: **Table S5.** Metabolites enriched into twenty different metabolic pathways. (XLSX 11 kb)

Additional file 7: **Table S6.** Comprehensive networks of metabolites and transcripts, changed in common KEGG pathways and differentially expressed genes among these pathways. (XLSX 13 kb)

Additional file 8: **Table S4.** Entire data set for metabolite profiling from different treatment soybean roots. (XLSX 10 kb)

## Abbreviations

4CL: 4-coumarate: coenzyme Aligase
ACC: 1-Aminocyclopropatie-1-carboxylate
ACO: 1-Aminocyclopropatie-1-carboxylate oxidase
ACOT: Acyl-CoA thioesterase
ACS: 1-Aminocyclopropatie-1-carboxylatesynthase
bifunctional AK-HSDH: bifunctional enzymes aspartokinase-homoserine dehydrogenase
CAD: Cinnamyl alcohol dehydrogenase
CYP: Cytochrome P450
GC-MS: Gas chromatography-mass spectrometry
ISR: Induction of systemic resistance
MGL: Methionine gamma-lyase
OPLS-DA: Orthogonal projections to latent structures-discriminate analysis
PAL: Phenylalanine ammonia-lyase
PGPR: Plant growth-promoting rhizobacteria
POD: Peroxidases
SCN: Soybean cyst nematode
VIP: Variable importance projection
