# Supplementary figures and images for: Transcriptomic and metabolomic analyses reveal that bacteria promote plant defense during infection of soybean cyst nematode in soybean
Source: BMC Plant Biol. 2018 May 11;18:86. doi: 10.1186/s12870-018-1302-9 (PMC5948838; doi:10.1186/s12870-018-1302-9)

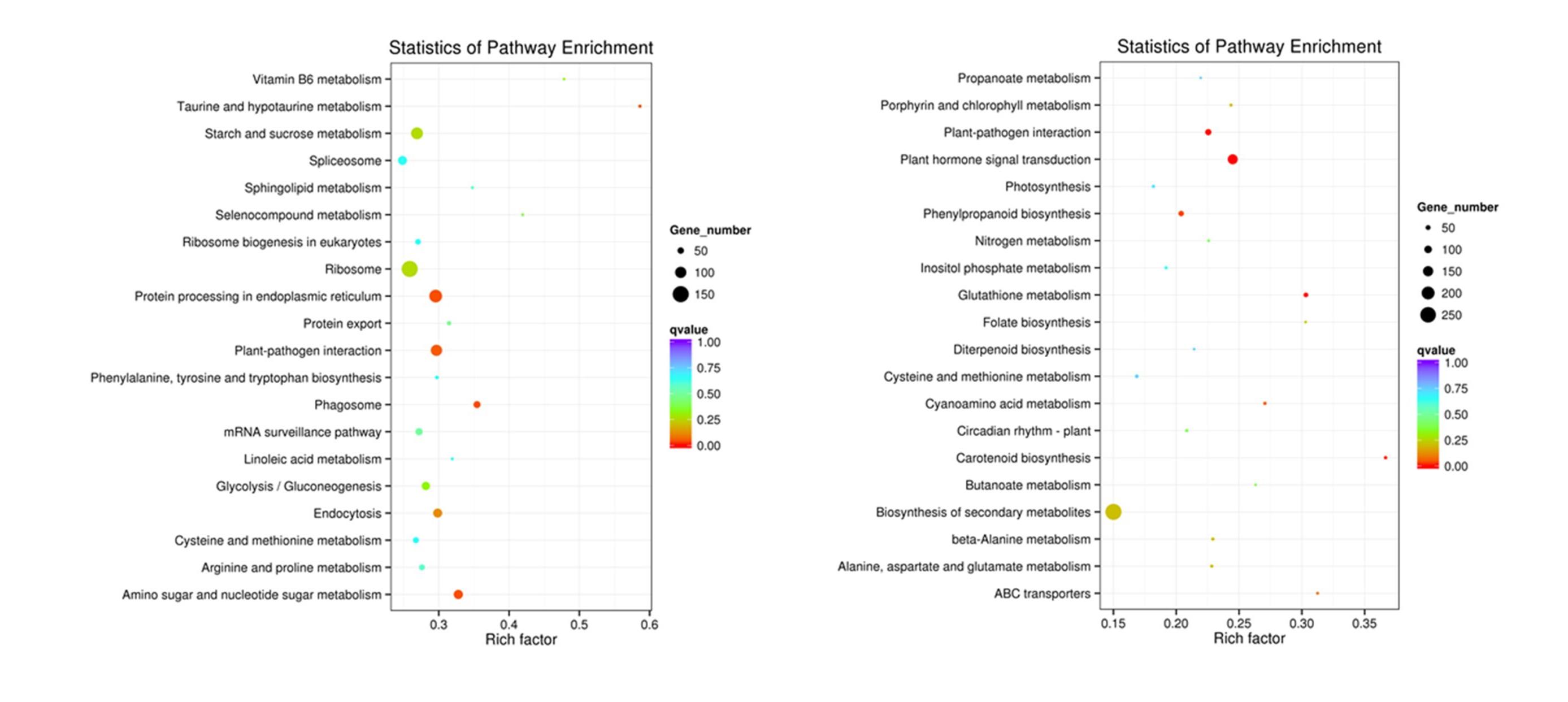

Supplement: Supplementary file 4 — Figure S1. Differentially expressed genes enriched KEGG pathway in CI vs CN group. (JPG 172 kb) [file 12870_2018_1302_MOESM4_ESM.jpg]

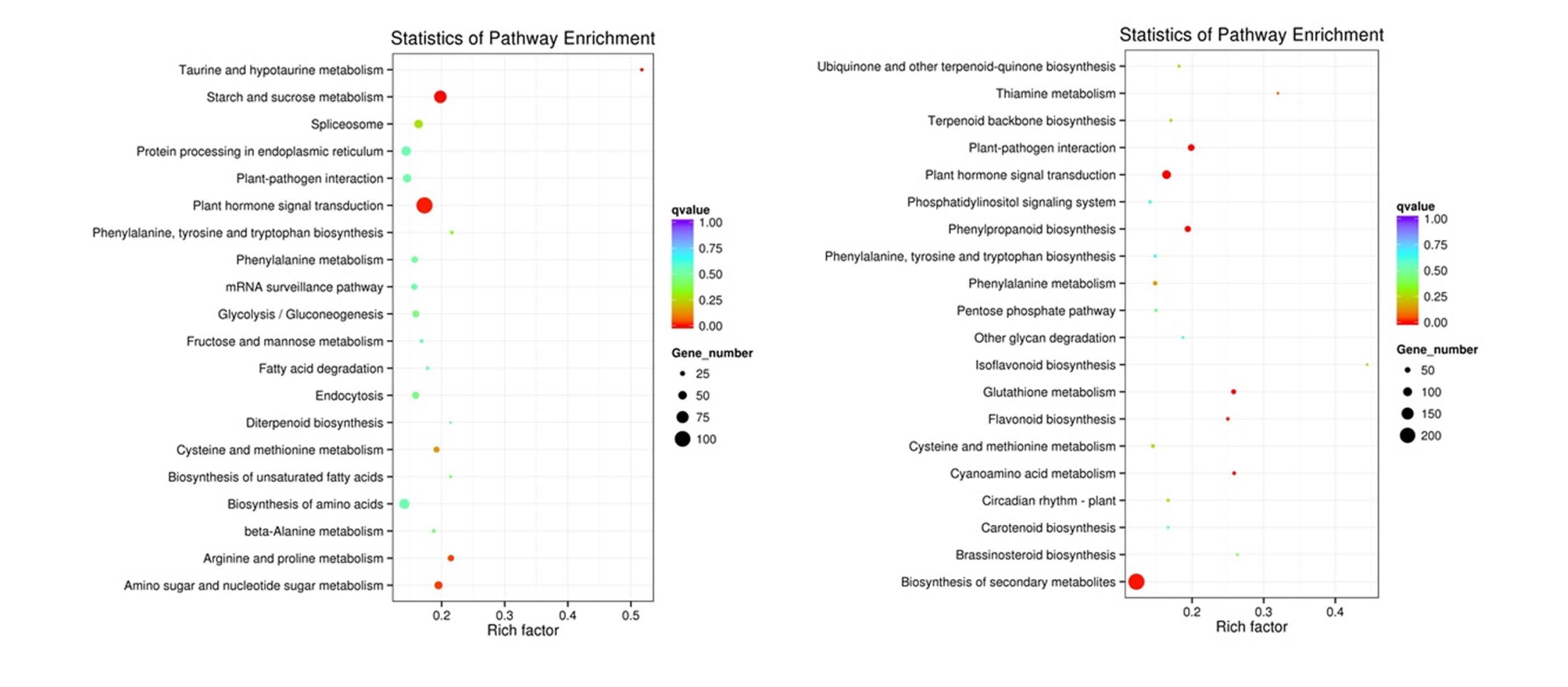

Supplement: Supplementary file 5 — Figure S2. Differentially expressed genes enriched KEGG pathway in TI vs TN group. (JPG 176 kb) [file 12870_2018_1302_MOESM5_ESM.jpg]
